# Enhancement of Luminescence of PET Films after Swift Heavy Ion Irradiation

**DOI:** 10.3390/polym15040910

**Published:** 2023-02-11

**Authors:** Adil Z. Tuleushev, Fiona E. Harrison, Artem L. Kozlovskiy, Maxim V. Zdorovets

**Affiliations:** Engineering Profile Laboratory, L.N. Gumilyov Eurasian National University, Astana 010008, Kazakhstan

**Keywords:** PET films, swift heavy ion, induced ordering, intrinsic luminescence, Urbach base line

## Abstract

The novelty of the study is that the ordering that occurs in a PET film under the action of SHI irradiation manifests itself as an increase in the integral intensity of intrinsic luminescence. The Urbach behaviour of the red shift of the absorption edge is used as a baseline for further analysis of experimental optical transmission spectra of PET films irradiated by swift heavy ions (SHI) previously published by the authors. Negative deviations of the experimental spectra from the Urbach baseline in the visible and UV parts of the spectrum are attributed to enhanced by SHI irradiation intrinsic luminescence. The observed dependence of the integral intensity of luminescence of irradiated PET films on the SHI fluence and ion charge provides further confirmation of the presence of SHI-induced ordering of the molecular structure in SHI latent tracks.

## 1. Introduction

The study of the properties of latent tracks in polyethylene terephthalate (PET) films of irradiated with swift heavy ions (SHI) continues to be the focus of attention of many scientific groups [1,2,3,4,5,6]. This is in part due to the relatively recent production of SHI-irradiated and UV-treated PET films whose permeability and selectivity is comparable to biological membranes [7,8]. In [7,8] the authors attribute the high permeability of such membranes to the presence of nanopores with a central pore diameter of less than 1 nm, and the high ionic selectivity to the electrostatic interaction between the partially dehydrated ions and the negatively charged subnanopore walls. The formation of nanopores along the center of the latent tracks is explained by the influence of the UV exposure on the irradiated PET film and subsequent immersion in the electrolyte, which washes out the volatile products of SHI-induced radiolysis and UV-induced photo-destruction from the latent track to form the nanopore. The negative charge on the nanopore walls is attributed to the formation of a thin layer of negative COO ions as a result of the interaction of CO and OH groups.

This explanation of the negative charge is difficult to reconcile with the underlying *δ*-electron model assumed by the authors. In [9], an analysis of X-ray diffraction studies of polyimide foil irradiated with swift U ions led the authors to conclude that there is a significantly electron-depleted region along the latent track axis, with a radial size of about 3 nm. In order to form a layer of negative charge on the subnanopore walls, the layer of COO ions would need to provide a negative charge above that needed to neutralize this positively charged region, which is not plausible within such a constrained geometry. Furthermore, transmission electron microscopy reported in [10] showed the presence of a region of altered density and the presence of an open channel along the axis of the SHI latent tracks. Since none of the mechanisms used to explain the formation of latent tracks (such as ion explosion spike [11] and the thermal spike [12,13,14]) predict any dilatation effects such as the disorder/order transition when SHI passes through a polymer film, the authors of [10] attributed the specified change in polymer density to the formation of gaseous and volatile products in the central part of the latent tracks and their subsequent removal through the open ends of the latent tracks.

We have previously found evidence that an increased density of carboxyl groups on the nanopore walls can exist before UV exposure and immersion in an aqueous electrolyte, as a result of SHI-induced ordering of the molecular structure of PET [15]. We used X-ray analysis to show the emergence of stable structural changes of the disorder/order type in the amorphous part of PET films under SHI irradiation. We demonstrated that the degree of ordering depended on both the SHI fluence and on the initial ion charge and involved both intra- and inter-molecular ordering induced by the residual electric field in the SHI latent tracks. This dependence on the ion charge, together with our further investigations [16,17], cannot be understood by considering only the swift *δ*-electrons ejected from the core of the latent track, but can be by considering also the slow electrons drawn into the core by the field of the passing ion [18].

The net result of these electron movements together with the electret properties of PET film [19] is a stable negative charge along the core of the latent track and a positively charged peripheral shell around it. The resulting radial electric field acts on dipole groups in the PET molecules, which rotate to align with the field gradient. This leads to the increased ordering, and changes in the distances between dipole groups. The study of optical effects that are sensitive to changes in distance between dipole groups should therefore provide additional information about the ordering of PET molecular structures in SHI latent tracks.

One such effect is the photoluminescence of PET films [20,21,22,23,24], which is sensitive to changes in the orientation of repeat units in the amorphous part of PET films [21,24]. In these studies, it was found that an increase in the ordering of the molecular structure of PET under one- or two-axis stretching and decrease in the distance between the dipole groups of repeat units of neighbouring chain molecules led to a significant increase in the luminescence yield. The luminescence was shown to be of Stokes type, concentrated in a photon energy range of 2.7–3.8 eV, and of an intensity small compared to the intensity of the light passing through the PET film sample. This suggests that irradiated PET films might also exhibit an enhanced luminescence yield. In addition, the observed luminescence photon energy range coincides with that of the red shift of the PET absorption edge under SHI irradiation [16], suggesting that these two processes may be connected. The experimental transmission spectra of SHI-irradiated PET [16] together with our analysis in [17] provide an opportunity to test this conjecture.

Photoluminescence can be studied through both emission and excitation spectra [25,26]. To obtain excitation spectra, the intensity of the light emitted by the sample is recorded (preferably in a narrow wavelength range) as a function of the wavelength of the excitation light. To obtain the emission spectrum, the spectral composition of the emitted light is recorded across the photon energy range of interest when the sample is excited by light with a higher photon energy. Our experimental set up [16] is close to that used in [21] to obtain the intrinsic PET excitation spectrum, where the monochromatic incident light was provided by a xenon lamp spectrophotometer. Although we did not measure the spectral composition of the emitted light, it is nevertheless possible to obtain information about the presence of luminescence from our experimental data.

We have previously noted that in “absorbance” mode, our spectrophotometer actually measures “non-transmittance” (or overall attenuation of the intensity of the incident illuminating light) [17]. It does so by measuring transmitted light at all frequencies above the sensitivity of the photodiode, assuming the sample is not optically active and thus that all the light transmitted is still of the incident frequency. If luminescence is present, the recorded measurement will comprise both the transmitted (attenuated) monochromatic light and the integral of the luminescence spectral function (such as that in [21] (Figure 3)), and the sample will look less absorbing than it would have, had it been optically inactive.

In [17] we found that for incident photon energies in the region ~ 2.5–3.8 eV the shape of the red shifted absorption edge corresponds well to the Urbach rule. This Urbach behaviour is the result of passive interaction between the film and the incident light and is associated with the steady growth of extended conjugated systems in PET films under increasing SHI fluences. The Urbach rule is characterized by the logarithm of the non-transmittance coefficient lnα*(*v*) being a linear function of the illuminating photon frequency *v*. The fact that our experimental results are generally well approximated by this linear relationship shows that generally luminescence is small compared to the overall transmission. But deviations of the experimental graph *below* the Urbach line mean that more light than expected is being transmitted (i.e., detected by the photodiode) for that frequency of illuminating light. We can study these deviations for each of our samples by using the ideal Urbach straight line for lnα*(*v*) as a baseline [27] and subtracting this from the observed data to produce a difference function. A negative difference function means that the light is less attenuated than would be expected for a passive material: the integrated emission light intensity recorded by the photodiode has been enhanced—and the only physically acceptable known mechanism for enhanced emission is luminescence.

**Figure 1:**
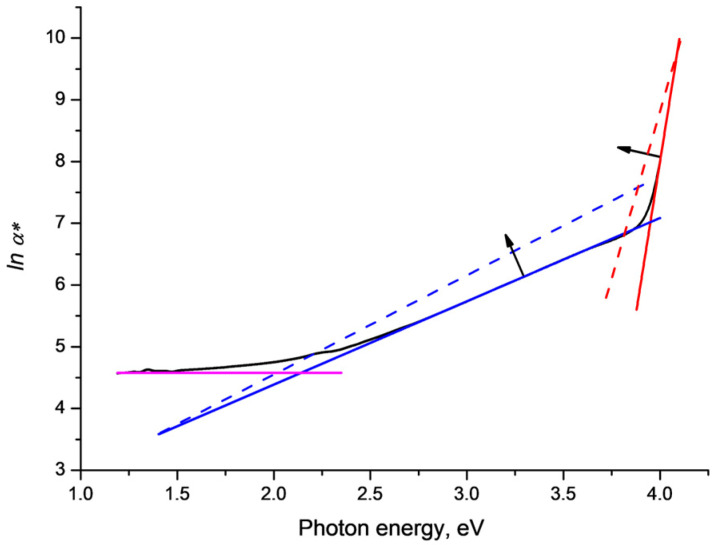
Linear approximations to the observed red shift of the absorption edge in PET films under SHI irradiation. Red and blue lines correspond respectively to the Urbach lines in the high-frequency UV and mid-frequency visible parts of the spectrum. Dotted lines and arrows show the general trend of rotation of these lines as the SHI fluence increases. The magenta line is an approximation in the low-frequency near-IR part of the spectrum where transmission is only very weakly dependent on the SHI fluence.

**Figure 2:**
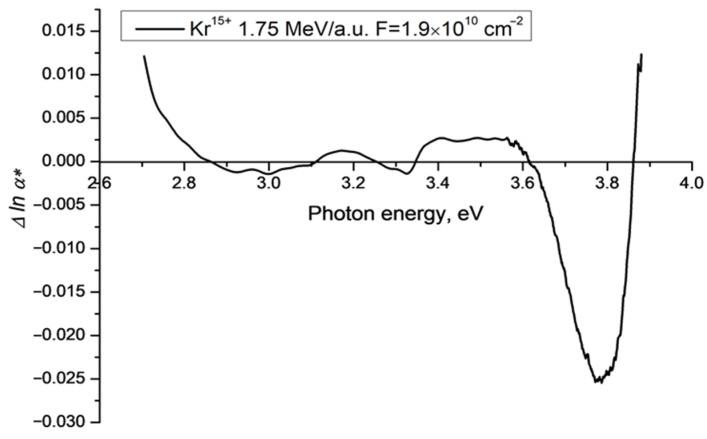
Difference function Δlnα* for a 12 μm PET film sample on a cylindrical aluminum drum irradiated with Kr^15+^ ions of energy 1.75 MeV/a.u. and a fluence of 1.9 × 10^10^ cm^−2^ in normal geometry [17]. The positive regions below 2.8 eV and above 3.9 eV correspond to the transition zones to other linear approximations as shown in Figure 1.

**Figure 3:**
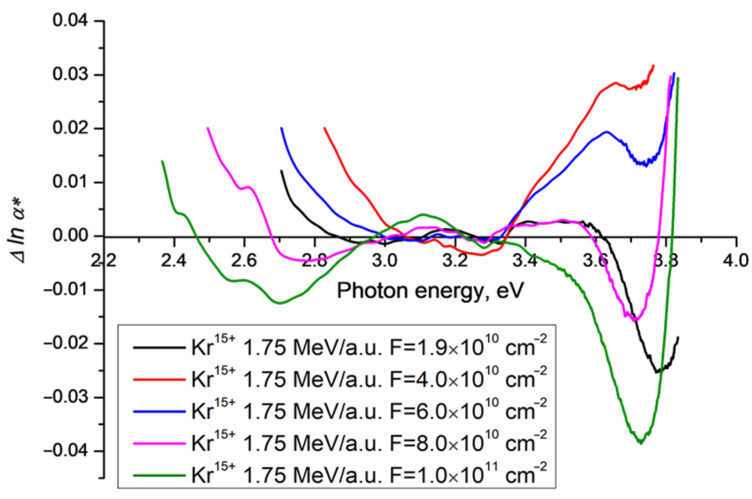
Difference functions Δlnα*(*ν*) for PET film samples irradiated on a cylindrical aluminum drum with Kr^15+^ ions of energy 1.75 MeV/a.u. and fluences ranging from 4 × 10^10^ cm^−2^ to 1 × 10^11^ cm^−2^ in normal geometry. The black line shows the same sample as in Figure 2.

**Figure 4:**
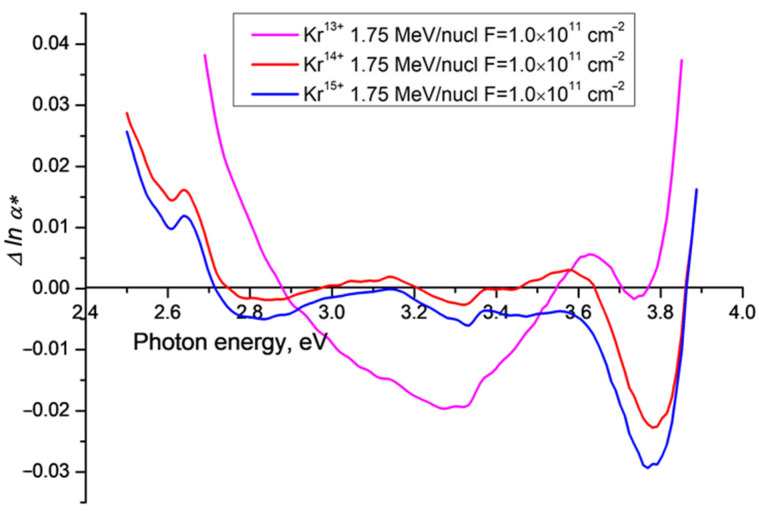
Difference functions Δlnα*(*ν*) for PET films irradiated with Kr ions of charge 13+, 14+ and 15+ with energy 1.2 MeV/a.u. and fluence of 1 × 10^11^ cm^−2^ at an angle of 42° to the normal.

**Figure 5:**
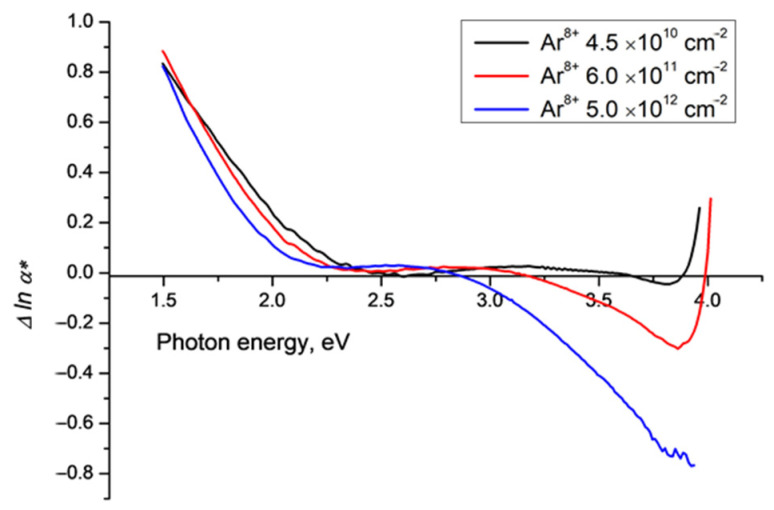
Difference functions Δlnα*(*ν*) for PET film samples irradiated with Ar^8+^ ions of energy 1.75 MeV/a.u. and fluences of 4.5 × 10^10^ cm^−2^, 6.0 × 10^11^ cm^−2^ and 5.0 × 10^12^ cm^−2^, at an angle of 42° to the normal. Note the *y* axis scale is an order of magnitude larger than in Figure 2, Figure 3 and Figure 4. The cut off in the UV region for the fluence of 5 × 10^12^ cm^−2^ (blue line) before the difference function returns to positive is due to experimental limitations on the number of data points in this region (for more details, see [16,17]).

The results we obtained in [17] can provide an illustration. As is well-known, the red shift of the absorption edge in PET films under SHI irradiation occurs in the UV/vis region. In the near IR region, transmission is high with almost no dependence on frequency. SHI irradiation has only a small effect and lnα*(*v*) has almost no dependence on *v*. In the red shift zone, the evolution of the absorption edge with growth in SHI fluence was found [17] (Figure 1, Figure 2, Figure 3, Figure 4 and Figure 5) to be well approximated by the rotation of two intersecting inclined straight lines. In the visible region the line rotates counterclockwise around a focal point in the energy range of 1.3–1.5 eV as the SHI fluence increases. In the UV region, the line is significantly steeper and rotates clockwise around a focal point in the energy range of 4.1 eV as the SHI fluence increases.

Figure 1 below is a schematic representation of the function lna*(*v*) (plotted against photon energy eV) for a 12 μm PET film sample on a cylindrical aluminum drum irradiated with Kr^15+^ ions with an energy of 1.75 MeV/a.u. in normal geometry and a fluence of 1.9 × 10^10^ cm^−2^ (black line). The magenta, blue and red straight lines show linear approximation trends in the near IR, visible and UV spectral regions, respectively. These ‘ideal case’ lines provide a theoretical lower limit on the attenuation of light passing through passive SHI-irradiated PET film samples.

## 2. Experiment

All experiments were carried out at the DC-60 heavy ion accelerator in Astana, Republic of Kazakhstan (INP, Astana, Kazakhstan) using PET films Hostaphan Mitsubishi Polyester Film RNK12 (Mitsubishi Polyester Film GmbH, Wiesbaden, Germany) with a thickness of 12 μm, and have been described in detail in our previous articles [15,16]. For ease of reading, the SHI irradiation parameters for the experimental results discussed below are given in the captions to the corresponding figures. Optical transmission spectra of irradiated and pristine PET films were obtained using the Jena Specord-250BU analytical spectrophotometer (Analytik Jena, Jena, Germany). The method of separating the smooth transmission curve from the interference fringes in the experimental transmission spectra is due to [28,29] and described in detail in [16].

## 3. Comparison of Spectral Dependences of lna*(*v*) with Urbach Approximations for Various Fluences and SHI Chargess

Figure 2 shows the difference function Δlna*(*v*) between the experimental values of the magnitude of lna*(*v*) and the Urbach baseline in the visible region for the sample in Figure 1. In the region of photon energies 2.9–3.9 eV the difference function falls below zero, with minima at 3.0 eV, 3.3 eV and 3.8 eV, which coincide with the PET film triplet energy levels found in [20]. The minimum at 3.8 eV is about 2.5% of the baseline Urbach value. Outside this region (below 2.9 eV and above 3.9 eV) the difference function is positive, as can be seen easily in Figure 1.

Figure 3 shows Δlnα*(*v*) for experimental data discussed in [17] for samples of PET film irradiated with Kr^15+^ ions of energy 1.75 MeV/a.u. and a range of fluences from 1.9 × 10^10^ cm^−2^ (as in Figure 2) to 1 × 10^11^ cm^−2^.

All five samples are qualitatively similar, with regions of negative Δlnα*(*v*) and clear negative minima. Many of the quantitative details are fluence-dependent and cannot be directly compared here due to the relative distortion introduced by the baselining of each difference function on its idealized Urbach line, which rotate around focal points as the fluence increases, but not necessarily at constant speed. For some values of fluence (4 × 10^10^ cm^−2^ and 6 × 10^10^ cm^−2^) the difference function is only briefly negative around 3–3.3 eV, despite being negative over a wider range for both higher and lower fluences. This merits further investigation but we suggest that this reversal in the behaviour of Δlna*(*v*) may be due to the fact that, as the SHI fluence increases, the area of unaffected pristine PET film (for which there is no absorption edge red shift effect) decreases, so the overall area of high transmission decreases, counteracting the enhanced transmission seen at lower fluences. With high enough fluence, the latent tracks begin to overlap [9,15,30,31,32,33], leaving no areas of pristine film, and Δlna*(*v*) returns to being negative.

For all fluences, the functions Δlnα*(*v*) show minima of approximately the same amplitude at a photon energy around 3.3–3.4 eV eV. While all experimental graphs show local minima at around 3.7–3.8 eV, their amplitude varies, with the most negative minima (occurring at the highest fluence) having a magnitude of about 4% of the baseline value. The minima at the lowest photon energy, which for a fluence of 1.9 × 10^10^ cm^−2^ occurs at about 3 eV (seen more clearly in Figure 2), disappears for intermediate fluences before reappearing at lower photon energy and with greater amplitude as the fluence increases further. At the maximum fluence of 1 × 10^11^ cm^−2^, this minimum is at photon energy of 2.7 eV with an amplitude of about 1% the baseline Urbach value. Taking the results from [21,24] together with [15,16], the behaviour of Δlna*(*v*) suggests that the observed excess light intensity, which is equal to the integral luminescence intensity, is associated with the process of ordering of the molecular structure of the PET film under the influence of SHI irradiation.

The dependence of Δlna*(*v*) on ion charge is shown in Figure 4, using the experimental data reported and discussed in [15,17] for Kr 13+/14+/15+ ions. As in Figure 3, all samples demonstrate qualitatively similar regions of negative Δlnα*(*v*), but with quantitative differences. For the PET sample irradiated with Kr^13+^ ions, there is a well-defined broad band minimum in the photon energy range 2.9 eV to 3.5 eV, resulting from the merging of peaks at 3.0 eV and 3.3 eV, with a maximum amplitude at about 3.3 eV of about 2% of the Urbach baseline. As before, there is a local minimum at about 3.7 eV.

For the higher ion charges of 14+ and 15+ the minima at 3.7 eV become more pronounced and more negative. The broad band observed for Kr^13+^ has separated into two negative minima at about 3.3 eV and 2.7 eV (as observed in Figure 3), with amplitudes of about 0.5% of the baseline.

Figure 5 shows Δlna*(*v*) for the observed spectra reported and discussed in [16,17] for PET film samples irradiated with Ar^8+^ ions in normal geometry and fluences up to 5 × 10^12^ cm^−2^. This is over an order of magnitude higher than the highest fluence used in our Kr ion experiments and as expected we see enhanced amplitude of effects.

The amplitude of the minimum in Δlna*(*v*) at around 3.8 eV for the lowest Ar fluence of 4.5 × 10^10^ cm^−2^ (black line) is of the same order (a few percent of the baseline) as that seen for similar Kr ion fluences. At the higher fluences of 6 × 10^11^ cm^−2^ and 5 × 10^12^ cm^−2^ the position of the minimum remains at 3.8 eV but the negative amplitude becomes exceptionally large, with Δlna*v equal respectively to −0.3 and −0.7 of the value of the Urbach baseline. These logarithmic ratios correspond to excess experimental light intensities of 30% and 50% above the Urbach line of the absorption edge of SHI irradiated PET films.

Our experiments span almost two orders of magnitude of SHI fluences (from ~10^10^ cm^−2^ to 10^12^ cm^−2^) across which the difference function Δlna*(*v*) shows over an order of magnitude change in the amplitude of the minimum at 3.8 eV (0.025–0.7). This approximately linear relationship with fluence suggests that the luminescence caused by SHI irradiation depends on the number of latent tracks.

Based on the above analysis of our previous experimental results we conclude that:

(1) SHI irradiation of PET films leads to induced luminescence along with a red shift of the absorption edge, in the photon energy range of 2.5–3.9 eV;

(2) this luminescence increases with an increase in the density of latent tracks (in line with the well-known dependence of luminescence intensity on the number of luminescence centers [25,26];

(3) the structure of triplet levels characteristic of the optical spectrum of a repeat unit of a PET chain molecule manifests itself in the spectral dependence of the integral induced luminescence.

## 4. Discussion of the Results

The photophysical studies of fluorescence in PET films reported in [20,21] provide an understanding of the molecular basis of the SHI-induced luminescence that we have observed. In [20], the authors investigate PET emission spectra at a molecular level, by comparing experimental observations of polarized and unpolarized emission spectra with computations based on a model of a single repeat unit (monomer) of the PET chain molecule. This monomer model is justified by the fact that the *π* − π* systems located on neighbouring terephthalate moieties in the PET chain molecule do not interact with each other because of the ethylenic groups separating them, so the electronic properties of PET are highly localised.

Many of the features of the experimental emission spectra in [20] are accounted for by this model, based on which the authors attribute the fluorescence peak at 338 nm (3.7 eV) to emission from the first singlet *π* − π* state S_1_ to the ground state S_0_. They assign the strong phosphorescence peak at 464 nm (2.7 eV) to the first excited triplet state and suggest that the density of the triplet states at 3.0 eV, 3.3 eV and 3.7 eV may make the probability of intersystem crossing comparable to or higher than the probability of fluorescence, thus explaining the high intensity of this peak relative to the fluorescence peaks. The authors note that fluorescence peaks at 368 nm (3.4 eV) and 564 nm (2.2 eV) cannot be accounted for by the monomeric model and suggest that these may be associated with (respectively) the ground and triplet states of a dimer composed of terephthalate moieties of repeat units of neighbouring PET chain molecules, which are not isolated from each other by ethylenic groups in the way neighbouring units in the same PET chain molecule are.

In [21], the authors used the intrinsic luminescence of semi-crystalline PET films to study changes in molecular orientation under the action of uniaxial and biaxial deformation (stretching) at temperatures above the glass transition. As part of their investigation, they conducted a photostationary fluorescence characterization of unoriented PET film, by taking the emission spectrum for wavelengths of excitation light across the range 260 nm to 360 nm in steps of 10 nm. This characterization demonstrated Stokes type fluorescence emission concentrated in the wavelength range 320–460 nm (2.7–3.9 eV).

Based on their observations and analysis, the authors conclude that the fluorescence sources are in the amorphous phase, and that deformation of the film results in both growth of the crystalline phase and increased ordering (of some kind) in the amorphous phase. This increases the formation of (unidentified) traps associated with fluorescence in the 360–420 nm range and leads to an increase in the intensity of the luminescence of the PET film induced by the deformation ordering. The authors also found confirmation that light absorption occurs in the terephthalate moieties, with two possible outcomes: re-emission of a photon with lower energy by the absorbing moiety; or migration of the exciton and capture by a trap within the amorphous phase, from where a photon with lower energy can be re-emitted.

Although our experimental setup was not designed to study luminescence, it shares enough with that used in [21] for the analysis therein to provide a qualitative explanation of the spectral dependencies presented in Figure 2, Figure 3, Figure 4 and Figure 5 above. In our experiments, spectra were built up in the same way as in [21], by stepwise increments in the wavelength of incident light. But instead of measuring the emission spectrum for each incident wavelength as a function of wavelength, the photodiode in our spectrophotometer records an emission intensity integrated over all wavelengths for each incident wavelength, and attributes it to the wavelength of the incident light. The variation of the integral emission intensity with excitation wavelength can be seen in the three-dimensional fluorescence characterization of a pristine PET film in Figure 3 in [21]. Unsurprisingly, the positions of the local minima in our difference functions (Figure 2, Figure 3, Figure 4 and Figure 5 above) correlate well with the fluorescence peaks found in [21].

Both [21] and our experiments show well-pronounced luminescence of PET films when illuminated with light with photon energy well below the energy of the allowed singlet-singlet transition (4.1 eV), which is the cause of the edge in pristine PET absorption spectra (see, for example, [20,21,34]). Since it is obvious that the PET film can emit only if it has previously absorbed, this confirms the existence of a significant singlet-triplet channel of absorption of light energy: although such transitions are forbidden in crystals, selection rules can be broken in amorphous systems [35].

The dependence of the intensity of the integral luminescence of PET films on the level of ordering of the molecular structure found in [21] is also confirmed by our results. The only difference is that in [21], ordering was achieved by stretching the PET film whereas in our experiments it was achieved by SHI irradiation.

As noted in [20,21] and supported by our studies, experimental observations cannot be explained within a monomer model and require consideration of the interaction between terephthalate moieties of repeat units of neighbouring PET chain molecules in the amorphous part to form larger systems (dimers or greater). In fact, the formation of dimers in the amorphous phase is possible even in the absence of any specific intervention to increase ordering since the distribution of rotation angles of terephthalate moieties relative to the axes of chain molecules is stochastic, so there will always be some that are sufficiently close for their dipole groups to form a conformational bond. Any intervention that increases the molecular ordering will reduce the average separation between neighbouring chain molecules and thus increase the likelihood of the formation of longer extended conjugated systems from terephthalate moieties of neighbouring chain molecules [36] and lead to an increase in luminescence [26].

Previously, we showed that SHI irradiation of PET films led to increased ordering in the latent tracks, due to the radial ordering of dipole groups of repeat units under the action of a residual cylindrical electric field [15]. This leads to the formation of spiral-shaped extended conjugated systems of mobile parts of repeat units in neighbouring chains, along the axis of the latent tracks, which are responsible for the red shift of the absorption edge PET films. The dimers discussed in [20,21] are the minimal case of an extended conjugated system formed as a result of interchain interaction between the dipoles of terephthalate moieties. We conclude that both the luminescence seen with increased ordering and the red shift of the absorption edge in PET are due to the growth of extended conjugated systems of identical terephthalate moieties of neighbouring chain molecules.

Since photons are absorbed by the terephthalate moieties, extended conjugated systems can absorb multiple photons. Within an extended conjugated system excited electrons can migrate from one terephthalate moiety to the next, and there is the possibility of interactions between excited electrons in adjacent moieties. One potential interaction mechanism is the Dexter mechanism of energy transfer via bilateral exchange of electrons [37,38,39], which we believe is responsible for the energy migration discussed in [21].

The Dexter mechanism can also result in triplet annihilation leading to two singlet states, with one electron in the ground state S_0_ and the other in an excited state lying above the first excited singlet state S_1_, of energy equal to the sum of the two triplet states [40,41]. The electron in this excited state can make a non-radiative transition to the first excited singlet state S_1_ followed by an allowed radiative transition to the ground singlet state S_0_. The probability of Dexter triplet annihilation depends on the density of excited triplet states. The extended conjugated systems along SHI latent tracks in PET films constrain the movement of excitations to essentially one dimension. This, together with the possibility of multiple simultaneous photon absorption by the many terephthalate moieties in the extended conjugated system, leads to a high density of triplet excitations and, consequently, to a high probability of their collision (triplet annihilation).

Such a mechanism could explain the significant integral luminescence intensity we have observed for incident photon energies well below the energy of the first excited singlet state S_1_. It is also possible that the strong increase in fluorescence observed for increased stretching of the PET film in [21] is based on the same pumping mechanism for the first excited singlet state.

## 5. Conclusions

The use of the Urbach line as a baseline makes it possible to study the detailed structure of the absorption edge of SHI-irradiated PET films. We attribute the negative deviations of the experimental data from this baseline to enhanced luminescence due to the SHI irradiation of the PET films.

The dependence of the luminescence of irradiated PET film on the SHI fluence is further confirmation of the presence of radiation-induced ordering of the molecular structure in SHI latent tracks.

The ordering induced by the cylindrically symmetric residual electric field along the axis of the SHI latent track provides an explanation for the observed change in density of the PET film as being due to dilatation, with bound carboxyl groups present on the walls of an open subnanopore at the core of the SHI trajectory before the irradiated film is immersed in an aqueous electrolyte. This, together with the results in [17] showing the presence of a net negative charge in the central part of the latent track, provide an alternative explanation for the remarkable experimental results obtained in [7,8].

## Data Availability

Not applicable.
